# A Few Charged Residues in Galectin‐3′s Folded and Disordered Regions Regulate Phase Separation

**DOI:** 10.1002/advs.202402570

**Published:** 2024-09-09

**Authors:** Yung‐Chen Sun, Tsung‐Lun Hsieh, Chia‐I Lin, Wan‐Yu Shao, Yu‐Hao Lin, Jie‐rong Huang

**Affiliations:** ^1^ Institute of Biochemistry and Molecular Biology National Yang Ming Chiao Tung University No. 155, Sec. 2, Linong St. Taipei 112304 Taiwan; ^2^ Taiwan International Graduate Program in Molecular Medicine National Yang Ming Chiao Tung University and Academia Sinica Taipei Taiwan; ^3^ Department of Life Sciences and Institute of Genome Sciences National Yang Ming Chiao Tung University No. 155, Sec. 2, Linong St. Taipei 112304 Taiwan; ^4^ Institute of Biomedical Informatics National Yang Ming Chiao Tung University No. 155, Sec. 2, Linong St. Taipei 112304 Taiwan

**Keywords:** cation‐π interaction, galectin‐3, intrinsically disordered proteins, liquid–liquid phase separation, NMR spectroscopy, prion‐like protein, protein evolution

## Abstract

Proteins with intrinsically disordered regions (IDRs) often undergo phase separation to control their functions spatiotemporally. Changing the pH alters the protonation levels of charged sidechains, which in turn affects the attractive or repulsive force for phase separation. In a cell, the rupture of membrane‐bound compartments, such as lysosomes, creates an abrupt change in pH. However, how proteins’ phase separation reacts to different pH environments remains largely unexplored. Here, using extensive mutagenesis, NMR spectroscopy, and biophysical techniques, it is shown that the assembly of galectin‐3, a widely studied lysosomal damage marker, is driven by cation‐π interactions between positively charged residues in its folded domain with aromatic residues in the IDR in addition to π–π interaction between IDRs. It is also found that the sole two negatively charged residues in its IDR sense pH changes for tuning the condensation tendency. Also, these two residues may prevent this prion‐like IDR domain from forming rapid and extensive aggregates. These results demonstrate how cation‐π, π–π, and electrostatic interactions can regulate protein condensation between disordered and structured domains and highlight the importance of sparse negatively charged residues in prion‐like IDRs.

## Graphical abstract

This study explores how galectin‐3′s intrinsically disordered and structured domains orchestrate phase separation through sparsely charged residues and pH response. Evolutionarily conserved charged residues in the structured domain mediate cation‐π interactions, which regulate π–π interactions from the disordered domain. Additionally, the disordered domain's sparse negative charges modulate self‐assembly and may prevent fiber aggregation, which can be a general trend in many prion‐like disordered proteins.

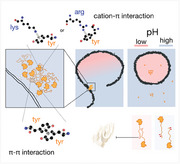

## 1 Introduction

Countless cellular biochemical reactions are precisely regulated in time and space. Membrane‐bound organelles, such as the nucleus, endoplasmic reticulum, and mitochondria, help to compartmentalize particular biochemical processes.^[^ ^1^ ^]^ Alternatively, many biological activities are orchestrated by compartments without lipid bilayers, known as membrane‐less organelles or biomolecular condensate,^[^ ^2^, ^3^ ^]^ dynamically assemble through the processes of phase separation.^[^ ^4^ ^]^ The condensation of biomolecules is well‐explained by the stickers‐and‐spacers model,^[^ ^5^, ^6^ ^]^ the stickers being structural domains or short motifs that promote intermolecular interactions, and the spacers, typically linker residues, enhancing the flexibility and solubility of these dynamic entities. Although some structured proteins can undergo phase separation,^[^ ^7^ ^]^ this is primarily a feature of intrinsically disordered proteins (IDPs) or proteins with intrinsically disordered regions (IDRs),^[^ ^8^ ^]^ which are widespread in the proteome,^[^ ^9^ ^]^ because of their unique combination of both stickers and spacers.^[^ ^10^ ^]^

The roles of stickers and spacers in phase separation are regulated by the equilibrium between attractive and repulsive forces. For example, the condensation of TDP‐43′s IDR hinges on the balance between hydrophobic attraction and repulsive electrostatic forces from its positive net charge.^[^ ^11^ ^]^ Similarly, the condensation propensities of the intracellular domain of nephrin,^[^ ^12^ ^]^ nucleophosmin,^[^ ^13^ ^]^ and G3BP1^[^ ^14^ ^]^ are governed by the interplay between positively and negatively charged residues in IDRs. The strength of attractive or repulsive electrostatic interactions can be modulated by post‐translational modifications: phosphorylation adding a negatively charged phosphate group to serine or threonine,^[^ ^15^, ^16^, ^17^, ^18^, ^19^ ^]^ acetylation neutralizing lysine's positive charge,^[^ ^20^ ^]^ and methylation eliminating the positive charge of arginine or lysine residues and increasing their hydrophobicity.^[^ ^16^, ^21^, ^22^ ^]^ These covalent modifications alter the electrostatic landscape and intricately adjust the balance of forces governing biomolecular condensation.

Apart from post‐translational modifications, pH variation in the cellular environment can alter the protonation states of charged amino acids, thereby influencing the proteins' conformational tendencies and hydration energies, as demonstrated in numerous model peptide and theoretical studies.^[^ ^23^, ^24^, ^25^ ^]^ Phase separation propensities can also be affected by variations in pH.^[^ ^26^, ^27^ ^]^ For instance, as the cytosolic pH in budding yeast becomes more acidic under starvation conditions, the glutamates in the prion protein Sup35's central IDR become protonated thereby reducing the repulsive forces acting against the assembly.^[^ ^28^ ^]^ However, while the global effects of cytoplasmic pH changes on phase separation are recognized, the effects of varying pH between subcellular compartments (due, e.g., to endocytic rupture) on biomolecular condensates have been relatively less explored.

A very recent study shows how densely charged amino acid tracts in proteins organize the nucleolar sub‐phases and create distinct physicochemical environments within the nucleus, particularly through pH gradients facilitated by protons recruited to these acidic amino acid tracts.^[^ ^29^ ^]^ On the other hand, a minimal number of charged residues could sufficiently influence the self‐association properties, just as critical as densely charged regions in mediating molecular condensation. Moreover, most IDRs are tethered to folded domains, and the interplay between the disordered and ordered regions is often overlooked. Galectin‐3 is a particularly well‐suited model to elucidate the relation between a few charged residues and their roles in response to pH changes because of its well‐documented self‐assembly mechanism^[^ ^30^, ^31^, ^32^ ^]^ and well‐established functions in experiencing pH‐variable environments:^[^ ^33^, ^34^, ^35^, ^36^ ^]^ It is related to endosome/lysosome integrity and autophagy regulation, particularly involved in damaged vesicles for autophagy through binding to β‐galactoside‐containing glycoconjugates on damaged vesicles. Moreover, galectin‐3′s ability to undergo endocytosis and participate in the intracellular sorting of glycoproteins is related to pH, which is significant in its roles in cellular trafficking and signaling pathways under different pH conditions.^[^ ^35^ ^]^ However, how galectin‐3 responds to different pH environments and the roles its sparsely populated charged residues play in the assembly‐related functions remain unclear.

**Figure 1:**
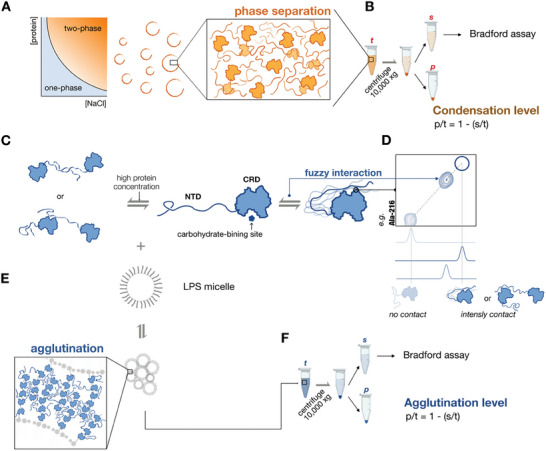
Schematic representation of galectin‐3 self‐association and assay techniques. A) Phase diagram highlighting the protein and NaCl concentration dependence of the boundary between galectin‐3 single‐phase (blue) and biphasic states (orange).^[^ ^31^ ^]^ NMR experiments were conducted under the one‐phase regime. B) The protein condensation levels (in the two‐phase regime) were quantified by determining the total protein concentration (*t*) and those in the supernatant (*s*) and pellets (*p*) after centrifugation. C) The intrinsically disordered N‐terminal domain (NTD) of galectin‐3 transiently interacts with the carbohydrate recognition domain (CRD) inter‐ and intramolecularly. D) In the one‐phase regime, the equilibrium level of NTD/CRD interactions can be determined by measuring chemical shift perturbations in NMR spectra, specifically from residues on the non‐carbohydrate‐binding face (using residue 216 as an example).^[^ ^30^ ^]^ E) Galectin‐3 agglutination ability was assessed using a lipopolysaccharide (LPS) micelle model. F) Agglutination levels were quantified by measuring galectin‐3 concentrations in supernatants (*s*) or pellets (*p*) relative to total protein concentration (*t*).

We have previously demonstrated that galectin‐3 undergoes phase separation under elevated protein or salt concentrations (the two‐phase regime; **Figure** **1**A).^[^ ^31^ ^]^ The tendency to assemble under specified conditions (different NaCl concentrations or pH conditions) is referred to as the “condensation level” (Figure 1B; detailed in Experimental Section). Under the one‐phase regime (in low protein concentration without salt), our NMR studies have also provided site‐specific insights, notably that the intrinsically disordered N‐terminal domain (NTD) interacts with the carbohydrate recognition domain's (CRD's) non‐carbohydrate binding face (with *f*ive β‐strands; *F*‐face) inter‐ and intramolecularly in a “fuzzy” manner (Figure 1C).^[^ ^30^ ^]^ This type of interaction between the NTD and CRD occurs within the fast‐exchange regime of NMR spectroscopy, enabling the detection of cross‐peaks from interacting residues, such as alanine 216, in the NMR ^1^H‐^15^N heteronuclear single quantum coherence (HSQC) spectra, which indicate a weighted equilibrium between intensely contacting ensemble and free states of the interacting regions (Figure 1D).^[^ ^30^ ^]^ Also under the one‐phase regime, galectin‐3 agglutinates (acting as a “bridge” to aggregate) glycosylated molecules. Lipopolysaccharide (LPS) is an often‐used model system to investigate galectin's agglutination ability.^[^ ^31^, ^37^, ^38^ ^]^ LPS forms micelle with its sugar moiety pointing outward (Figure 1E). By adding LPS micelles to induce functional agglutination (Figure 1E) and measuring the protein concentration in the supernatant after centrifugation by Bradford assay or SDS‐PAGE (Figure 1F; Figure S1, Supporting Information), we can define the “agglutination levels” between different constructs. Combining these assays on systematically designed galectin‐3 constructs, we elucidate the roles of the two negatively charged residues in the intrinsically disordered NTD and the positively charged residues on the structured CRD.

## 2 Results

### 2.1 Conservation of Charged Residue Distribution in Galectin‐3

**Figure 2:**
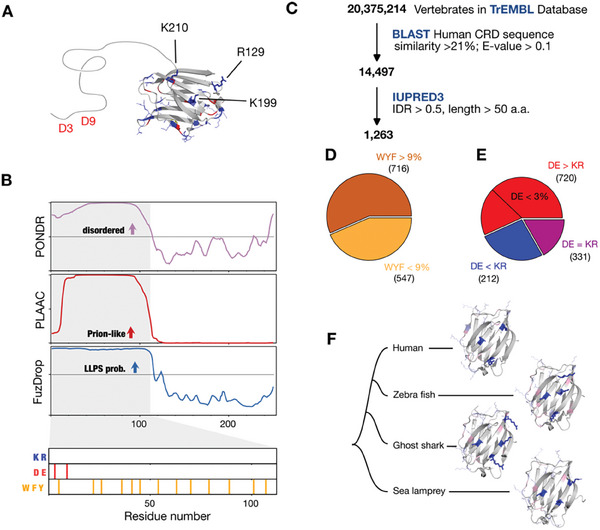
Bioinformatic analysis of galectin‐3. A) Charged residues are labeled on the crystallographic structure of the carbohydrate‐recognition domain (CRD; PDB: 2NMO): lysine/arginine (K/R) in blue and aspartate/glutamate (D/E) in red. Three positively charged residues, R129, K199, and K210, are highlighted on the non‐carbohydrate‐binding face (*F*‐face). The two negatively charged aspartates at positions 3 and 9 in the intrinsically disordered N‐terminal domain (NTD) are labeled in red. B) Structural disorder, prion propensity, and phase separation, respectively, are predicted by the PONDR,^[^ ^75^ ^]^ PLAAC,^[^ ^76^ ^]^ and FuzDrop^[^ ^55^ ^]^ algorithms. The NTD is shaded in grey, and the locations of positively charged (K, R), negatively charged (D, E), and aromatic (tryptophan [W], phenylalanine [F], tyrosine [Y]) amino acids are shown in the lower part of the panel. C) Bioinformatic analysis workflow with the number of protein sequences analyzed at each stage. D,E) Pie charts illustrating the proportions of the 1263 galectin‐like sequences with intrinsically disordered regions (IDRs) with (D) more or <9% of aromatic residues and (E) more, less or an equal number of positively‐ and negatively‐charged residues, with the subset with <3% of negatively charged residues also indicated. The values in parentheses are the number of sequences. F) AlphaFold2 predicted structures of galectin‐3′s CRD of the three most evolutionary distant vertebrates to humans.

Galectin‐3′s intrinsically disordered NTD has only two charged residues (Asp‐3, Asp‐9; both are negative) in humans and is predicted to have high prion‐likeness and phase separation propensity. Meanwhile, the CRD has three positively charged residues (Arg‐129, Lys‐199, Lys‐210) on its *F*‐face (**Figure** **2**A,B). This distribution prompts our inquiry into the role of these charged residues in mediating NTD/CRD interactions.

We first investigated the evolutionary conservation of charged residues in galectin‐3. The lack of structural elements limits the ability of multiple sequence alignment to identify conserved residues among IDRs.^[^ ^39^ ^]^ We therefore searched for sequence homologs of galectin‐3′s CRD in the vertebrate proteome (∼20 million sequences) and found ≈15000 matches (Figure 2C). We then used IUPRED3^[^ ^40^ ^]^ to evaluate their structural disorder content and identified 1263 sequences with at least one IDR of more than 50 consecutive residues (Figure 2C). More than half of these galectin‐3‐like proteins (57.9%; 716/1263) contain more than 9% of aromatic residues (the average content of the proteome as a whole:^[^ ^41^ ^]^ tryptophan, 1.5%; tyrosine, 3.5%; phenylalanine, 4.0%) in their IDRs (Figure 2D). An increased prevalence of aromatic residues is uncommon in IDRs,^[^ ^42^, ^43^ ^]^ but is observed in RNA‐binding proteins,^[^ ^44^ ^]^ suggesting potentially conserved functions,^[^ ^44^ ^]^ and our previous findings for human galectin‐3 suggest they have a functional role in agglutination.^[^ ^31^ ^]^ Additionally, 720/1263 sequences (57.0%) have IDRs with a net negative charge, of which nearly two‐thirds (480/720) have <3% of negatively charged residues (Figure 2E), compared with 12.5% in the proteome as a whole,^[^ ^41^ ^]^ which is striking because negatively charged residues are typically more prevalent in IDRs.^[^ ^42^ ^]^ The increased prevalence of aromatic residues and the low prevalence of negatively charged residues are expected, therefore, to be an evolutionarily conserved pattern in the IDRs of galectin‐3‐like proteins.

Since our search for homologs was based on human galectin‐3′s CRD, the structural domains of the 1263 identified sequences should be similar. To analyze the spatial charge distributions in these structural domains, we selected sequences from the three vertebrates most distantly related to humans (zebrafish, ghost sharks, and sea lampreys) and predicted their structures using AlphaFold2 (Figure 2F). Remarkably, the positively charged residues are located in similar positions on the *F*‐face in all these structures (Figure 2F). This similarity in charge distribution, alongside the NTD's conserved sequence patterns (Figure S2, Supporting Information; despite low sequence similarity), supports our hypothesis that these charged residues are functionally important.

### 2.2 The Positively Charged Residues in the Folded Domain Contribute to Intramolecular NTD/CRD Interactions through Cation‐π Interactions

**Figure 3:**
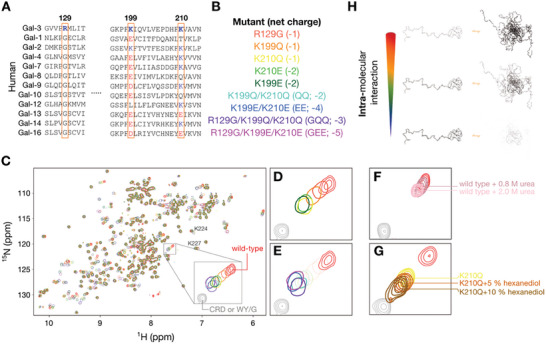
Analysis of intramolecular interactions between the N‐terminal domain (NTD) and the carbohydrate‐recognition domain (CRD). A) CRD sequence alignment between human galectins, with positively charged residues on the non‐carbohydrate‐binding face (*F*‐face) highlighted. B) List of mutations used in the study, with the corresponding net charge changes. C) Superimposed ^1^H‐^15^N HSQC spectra of the mutants and wild‐type galectin‐3, color‐coded as shown in panel (B). The large shift for residue 216, indicative of altered NTD/CRD interactions, is highlighted in panels D,E). F,G) ^1^H‐^15^N HSQC cross‐peak for residue 216 (F) in wild‐type galectin‐3 upon adding 0.8 or 2 M urea, and (G) in the K210Q mutant in the presence of 5–10% 1,6‐hexanediol. H) Schematic illustrations of the level of disruption of intramolecular NTD/CRD interactions associated with each mutation or achieved by adding chemical agents that interfere with hydrophobic contacts.

Although the positively charged residues on the *F*‐face are located similarly to those in other IDR‐carrying galectin orthologs (Figure 2F), their physical properties differ widely from those in the human galectin family (**Figure** **3**A). Residue 129 is arginine in galectin‐3, but glycine in the paralogs, and while residue 199 is lysine in galectin‐3, many of the paralogs have a negatively charged residue in this position. Residue 210 is more variable, with lysine in galectin‐3 and polar or negatively charged amino acids in the paralogs. These differences between galectin‐3 and its paralogs emphasize the importance of these charged residues for the different functions of the galectin family proteins.

To elucidate the roles of these charged residues, we systematically substituted the two lysines with either polar glutamine (K199Q, K210Q) or negatively charged glutamate (K199E, K210E), and replaced Arg‐129 with the more widely conserved glycine (R129G). These mutations were also combined pairwise (QQ, EE) or three‐way (GQQ, GEE; Figure 3B) to explore the effects of altering the overall charge content.

The overlay of ^1^H‐^15^N HSQC spectra of all the constructs in 40 µm sample concentration (one‐phase regime; Figure 3C) shows that the positively charged residues are indeed involved in the interaction equilibrium between the NTD and CRD. The cross‐peak from Ala‐216, on the *F*‐face but at a distance from the mutation sites, serves as the primary indicator for this interaction (Figure 3C–E). The point mutant with the most pronounced shift in Ala‐216 was K210Q, indicating that this mutation significantly disrupts NTD/CRD interactions (Figure 3D). Glutamate at position 199 (K199E) induced a more marked perturbation than glutamine (K199Q) did (orange and dark green in Figure 3D). In contrast, the corresponding substitutions at lysine 210 did not significantly alter the cross‐peak position (yellow versus light green in Figure 3D). These perturbations were enhanced in the double and triple mutants, but with similar peak positions in each case (Figure 3E).

We have shown previously that substituting all tryptophan and tyrosine residues in the NTD with glycine (WY/G) disrupts inter‐ and intramolecular interactions and phase separation, underlining the critical role of these aromatic residues.^[^ ^31^ ^]^ The attenuated NTD/CRD interactions observed upon removing charged residues from the CRD support the involvement of cation‐π interactions. However, the discrepancy between the effects observed in the double and triple mutants compared to the WY/G construct suggests that other forces are also involved at the NTD/CRD interface. Chemical shift perturbations were also observed for positively charged residues such as Lys‐224 and Lys‐227 (Figure 3C), hinting that these may be “fuzzy” sites for cation‐π interactions. Furthermore, adding urea at concentrations below 2 m (to avoid denaturing the protein) to wild‐type galectin‐3 or 5 ∼ 10% 1,6‐hexanediol to the K210Q mutant (in both cases to disrupt hydrophobic interactions)^[^ ^45^, ^46^ ^]^ led to chemical shift perturbations toward the positions observed in the least interacting conformation (Figure 3F,G; Figure S3, Supporting Information), indicating that hydrophobic interactions also contribute to these contacts. The charged residues on the *F*‐face of the CRD are thus implicated in intramolecular interaction between the NTD and CRD through cation‐π interactions, with additional contributions, at least from hydrophobicity (Figure 3H). We next examined whether these intramolecular interactions also affect intermolecular self‐association.

### 2.3 The Charged Residues on the F‐Face of the CRD Contribute to Self‐Association

**Figure 4:**
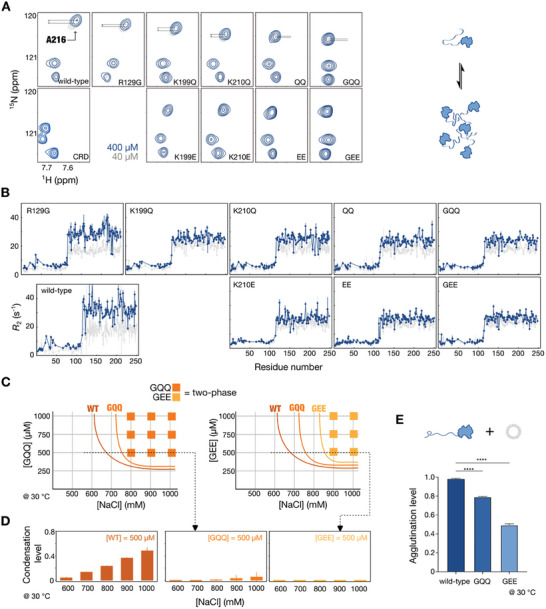
Influence of positively charged residues in the carbohydrate‐recognition domain (CRD) on intermolecular interactions. A) Alanine 216 region of ^1^H‐^15^N HSQC spectra showing the chemical shift changes between protein concentrations of 400 µm (blue) and 40 µm (gray), with the observed perturbations in the upper row indicated with gray lines. B) Transverse relaxation rate constants (*R*_2_) for wild‐type galectin‐3 and the mutants at concentrations of 400 µm (blue) and 40 µm (gray). C) Phase diagrams of GQQ (R129G/K199Q/K210Q) and GEE (R129G/K199E/K210E) constructs under different protein and NaCl concentrations. Solid squares represent proteins under such conditions showing condensates under the microscope. The phase boundary for the wild type (WT) is based on our previous work.^[^ ^31^ ^]^ D) Condensation levels for 500 µm WT, GQQ, and GEE constructs under various NaCl concentrations. E) Lipopolysaccharide agglutination assay is used to determine agglutination levels of these three constructs. Statistical analysis was performed using one‐way ANOVA followed by Dunnett's post‐hoc test (*n* = 3; ^****^ *p* < 0.0001).

To evaluate the influence of charged residues on galectin‐3 self‐association in the one‐phase regime, we compared NMR data at protein concentrations of 400 and 40 µm, thereby with a different equilibrium between monomeric and assembled forms.^[^ ^30^ ^]^ There was evidence of increased intermolecular NTD/CRD interactions at the higher protein concentration, as shown in chemical shift perturbations (**Figure** **4**A) in the wild‐type and the R129G, K199Q, and K210Q mutants, but much reduced in the others, similar to in the CRD‐only construct (Figure 4A; Figure S4, Supporting Information for the full spectra). We also measured transverse relaxation rate constants (*R*_2_), an NMR parameter sensitive to changes in molecular tumbling and weak self‐association^[^ ^47^, ^48^, ^49^ ^]^ under the two protein concentrations. *R*_2_ values typically increase with protein concentrations when the equilibrium shifts toward multimeric assemblies.^[^ ^47^, ^48^, ^49^ ^]^ Here, *R*_2_ values were higher at the higher protein concentration for all mutants, but the increase was weaker in the double‐ and triple‐mutants (Figure 4B).

In determining the thermodynamic parameters of condensation between the wild type and mutants, protein samples must remain reversible across a broad range of temperature and protein concentration conditions. This situation is achievable in some instances with IDRs without their structured domains or using model polypeptides.^[^ ^21^, ^50^, ^51^, ^52^, ^53^, ^54^ ^]^ However, our system contains a structured domain and aggregates at high concentrations (500 µm) or elevated temperatures (≈40 °C). Consequently, determining binodal in the [protein]/temperature phase diagram is impractical in our system. Therefore, we compared their assembly tendency under the condensation conditions (two‐phase regime) using the [protein]/[salt] phase diagrams of the wild‐type, GQQ, and GEE mutants (Figure 4C). The GEE constructs exhibited a higher threshold for condensation observed from the microscope than the GQQ and wild‐type samples. To mitigate the subjectivity of visual observations, we quantified the condensation levels (as illustrated in Figure 1B). At 500 µm samples with different salt concentrations, the condensation levels were lowest for the GEE mutant, higher for the GQQ mutant, and highest for the wild‐type (Figure 4D).

Finally, using LPS micelles as the functional assay (low protein concentration without salt, i.e., in the one‐phase regime), the mutant agglutinated less than the wild type, with 20% less agglutination for the GQQ construct and 50% less for the GEE mutant (Figure 4E). These decreases in agglutination are less marked than observed for the WY/G construct (Figure S1B, Supporting Information) and are consistent with the observed residual NTD/CRD intramolecular interactions, suggesting that interaction forces other than cation‐π interactions are involved (Figure 3F,G), including intermolecular π‐π interactions between NTDs.

Collectively, our data indicate that positively charged residues on the *F*‐face of the CRD are crucial for NTD/CRD interactions and the agglutination process. The observed reduction in self‐association and agglutination upon removal of these residues supports the existence of cation‐π interactions between the NTD and CRD. However, the persistence of residual interactions in the mutants suggests that additional mechanisms, such as π‐π interactions between NTDs and hydrophobic contacts between the NTD and CRD, also contribute to self‐association and agglutination. The next step of this study is to explore whether negatively charged residues also contribute to NTD/CRD interactions.

### 2.4 The Two Aspartates in the NTD Are Negative Regulators of Galectin‐3 Condensation and Are Not Involved in NTD/CRD Interactions

**Figure 5:**
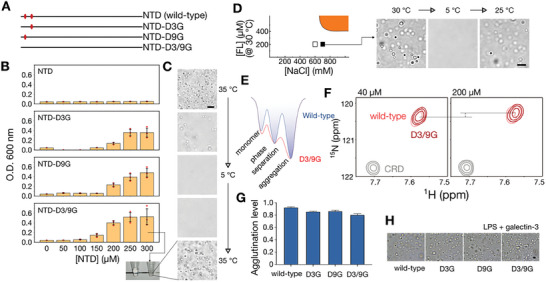
Characterizing the levels of self‐association and assembly of the N‐terminal domain (NTD) charge‐depleted mutants of galectin‐3. A) Schematic representation and nomenclature of the NTD‐only construct and mutants. B) Optical density measurements at 600 nm (O.D. 600 nm), indicating the turbidity of various concentrations of NTD‐only constructs. C) Temperature reversibility of 300 µm NTD‐D3/9G condensates under the microscope (scale bar: 10 µm). D) Micrographs (scale bar: 10 µm) showing that the full‐length D3/9G mutant condenses at a lower protein/NaCl concentration threshold than the wild‐type (phase diagram from our previous study^[^ ^31^ ^]^ shown in orange). No condensates were observed at 200 µm protein with 600 mm NaCl (open square). The condensates at 200 µm protein with 700 mm NaCl (filled square) were reversible but were irreversible aggregates at higher protein or salt concentrations. E) The energy landscape showing the lowered energy barrier between aggregation and the phase‐separated states of the D3/9G construct to explain the greater aggregation tendency of the D3/9G mutant. F) Alanine 216 region of the ^1^H‐^15^N HSQC spectra of wild‐type galectin‐3 (red), the D3/9G mutant (dark red), and the carbohydrate‐recognition domain (CRD)‐only construct (grey) at protein concentrations of 40 µm (left panel) and 200 µm (right panel). G) Lipopolysaccharide (LPS) agglutination assay of full‐length galectin‐3 and mutants. H) Bright‐field microscopic images of LPS with different galectin‐3 constructs before centrifugation (scale bar: 10 µm).

To clarify the role of the two aspartates in the NTD, they were replaced with glycine, initially in an NTD‐only construct, yielding the variants NTD‐D3G, NTD‐D9G, and the double mutant NTD‐D3/9G (**Figure** **5**A). In line with our previous observations,^[^ ^31^ ^]^ the wild‐type NTD does not condense at protein concentrations below 300 µm in the presence of 500 mm NaCl (Figure 5B). However, removing the aspartates decreases the concentration threshold for phase separation, as evidenced by optical density measurements at 600 nm (Figure 5B). The observed condensation is reversible in the manner of a lower critical solution temperature phase separation (Figure 5C). In other words, the repulsive force from the rare but evolutionarily conserved negative charges in the NTD increases the concentration threshold for condensation.

In full‐length galectin‐3, the D3/9G variant also underwent phase separation at a lower protein concentration threshold than the wild‐type did (Figure 5D). However, as a general metastable feature of many proteins’ phase separation^[^ ^55^ ^]^ (Figure 5E), this mutant has a lowered energy barrier to aggregate from the phase‐separated state and thus is more prone to aggregate. Accordingly, we kept the protein concentration below 200 µm to avoid irreversible aggregation, thereby limiting the range of potential differences in *R*_2_ values between the wild type and mutants (Figure S5A, Supporting Information). Intriguingly, there were no significant chemical shift perturbations in *F*‐face residues implicated in NTD/CRD interactions (e.g., Ala‐216) between the D3/9G mutant and the wild‐type (Figure 5F; Figure S5B, Supporting Information). This is different from what was observed for mutation sites on the *F*‐face (Figure 3C), suggesting that removing the two aspartates does not noticeably alter interactions between the NTD and the *F*‐face of the CRD.

LPS assays showed that these mutants had a marginally lower tendency to agglutinate compared with the wild type (Figure 5G), as reflected also by similar crowdedness under the microscope (Figure 5H). The D3/9G mutant's increased phase separation propensity is therefore independent of its tendency to agglutinate. This discrepancy between the increased phase separation propensity observed for the D3/9G mutant and its slightly lowered agglutination propensity could be due to differences in NaCl concentrations between the phase separation and LPS assays. It is also possible that the LPS agglutination capacity was nearly maximized for the wild type, such that any further enhancements in LPS agglutination in the mutants could not be discernible.

The insights from the above studies are that the two aspartates do not significantly affect NTD/CRD interactions but negatively regulate NTD/NTD association in providing electrostatic repulsion. Because galectin‐3 is often found in areas of the cell prone to fluctuations in pH, such as the vicinity of damaged lysosomes,^[^ ^33^, ^34^, ^35^ ^]^ and pH variations affect the protonation states of charged amino acid side chains, we next set the stage for a deeper exploration of how pH influences galectin‐3′s functional dynamics and assembly mechanisms.

### 2.5 Galectin‐3 Shows a Stronger Assembly Tendency at Lower pH

**Figure 6:**
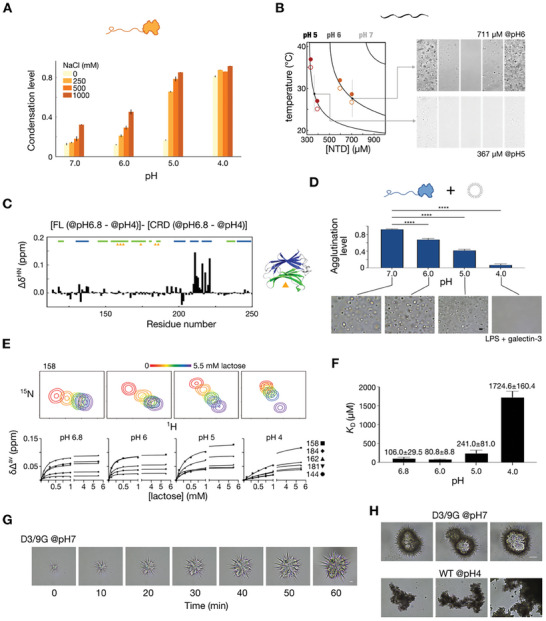
pH‐dependent phase separation and agglutination propensity of galectin‐3. A) Condensation levels of galectin‐3 under different pH conditions with different NaCl concentrations. B) Phase separation for the N‐terminal domain (NTD)‐only construct of galectin‐3 at various pHs. Closed and open circles represent the conditions at which condensates were or were not observed by microscopy (Figure S6, Supporting Information). Reversibility was observed for the NTD‐only construct at pH 6 (711 µm) and pH 5 (367 µm) under the conditions indicated on the phase diagram. C) Residue‐specific chemical shift perturbation (Δδ^HN^) for full‐length galectin‐3 between pH 6.8 and pH 4, normalized to the perturbation measured for the same pH change in the carbohydrate recognition domain (CRD)‐only construct. D) Lipopolysaccharide agglutination assay of galectin‐3 at different pHs with corresponding pre‐centrifugation micrographs. Statistical analysis was performed using one‐way ANOVA followed by Dunnett's post‐hoc test (*n* = 3; ^****^ *p* < 0.0001). E) Measured dissociation constants (*K*_D_) for CRD/lactose binding at different pHs, as exemplified by chemical shift perturbations at residue 158. The fits for five residues in the lactose binding site are presented. F) Calculated *K*_D_ values for CRD/lactose interactions at the studied pHs. G) Time‐lapse bright‐field microscopic images of the aggregation process for the D3/9G mutant at pH 7 (scale bar: 10 µm). H) Representative bright‐field microscopic images comparing the aggregate morphologies of the D3/9G mutant at pH 7 (upper row) and of wild‐type galectin‐3 at pH 4 (lower row), highlighting the reproducibility and distinctive characteristics of the two types of aggregates.

Full‐length galectin‐3 exhibited a greater tendency to aggregate at lower pH conditions, similar to the D3/9G construct depicted in Figure 5E. This aggregation complicates concentration‐dependent NMR experiments at low pH. Despite this challenge, galectin‐3′s condensation levels increased under low pH conditions across various NaCl concentrations (**Figure** **6**A). However, these condensates were not consistently reversible with temperature changes. On the other hand, the NTD‐only construct demonstrated higher solubility at low pH and demixed at lower thresholds. These condensates were reversibly affected by temperature changes at pH 5 and 6 without salt (Figure 6B; Figure S6B, Supporting Information). These findings align with the reduced repulsive force due to the partial protonation of aspartates in the NTD.

The ^1^H‐^15^N HSQC spectra of full‐length galectin‐3 and the CRD‐only constructs during titration from pH 6.8 to pH 4 are shown in Figure S6A (Supporting Information). Chemical shift differences were compared with those observed between pH 6.8 and pH 4 in the CRD‐only construct to eliminate perturbations caused by pH differences (Figure 6C). This differential analysis revealed that the most substantial perturbations occur on the *F*‐face, suggesting that NTD/CRD interactions are sensitive to changes in pH (Figure 6C). A plausible explanation is that the enhanced NTD/NTD association (because of reduced electrostatic repulsion) attenuates the NTD/CRD interaction.

### 2.6 The Agglutination Ability of Galectin‐3 Is Reduced at Acidic Conditions Due to the Reduced Binding Affinity

Although the self‐association propensity increased at lower pH, agglutination in LPS assays decreased (Figure 6D). Despite maintaining similar sample crowdedness in buffers at pH > 5, no assemblies of full‐length galectin‐3 and LPS were observed under the microscope at pH 4 (Figure 6D). This pronounced decline in agglutination is attributed to the decreased binding affinity of galectin‐3′s CRD, as confirmed by NMR titration experiments with lactose across different pH conditions, which showed that reduced binding affinity correlated with the decrease in pH (Figure 6E,F; Figure S6C, Supporting Information), in agreement with previous fluorescence anisotropy results.^[^ ^56^, ^57^ ^]^

### 2.7 The Roles of Two Negatively Charged Residues in the NTD

Galectin‐3′s reduced binding affinity at low pH counterbalances its increased propensity to self‐assemble, presenting an apparently counterintuitive scenario in which the role of negative charges in regulating assembly appears superfluous at low pH. However, the scenario at low pH differs from that of the D3/9G mutant since the presence of the side chains' partially protonated carboxyl groups provides repulsive forces that mitigate assembly. The aggregates of full‐length galectin‐3 at pH 4 and of the D3/9G mutant at pH 7 had different morphologies (Figure 6G). For D3/9G, we observed “sea urchin” or “starburst” shapes (Figure 6H), which grow over time but not in the instance of the aggregates of the wild‐type at acidic pH (Figure S7, Supporting Information). The morphology of D3/9G mutants is similar to those reported by Patel et al. for an amyloid‐disease mutant of FUS protein.^[^ ^58^ ^]^ FUS protein is a widely studied system for the relationships between amyloid formation and phase separation,^[^ ^59^, ^60^ ^]^ which shares with galectin‐3′s NTD a scarcity of negative charges and multiple aromatic residues that promote phase separation via π‐π or cation‐π interactions with its arginine‐glycine‐rich domain (Figure S8A, Supporting Information).^[^ ^22^, ^61^ ^]^ Therefore, NTD's two charged residues likely regulate its assembly tendency and prevent rapid fiber formation driven by the prion‐like nature of this region (Figure 2B).

## 3 Discussion and Conclusion

**Figure 7:**
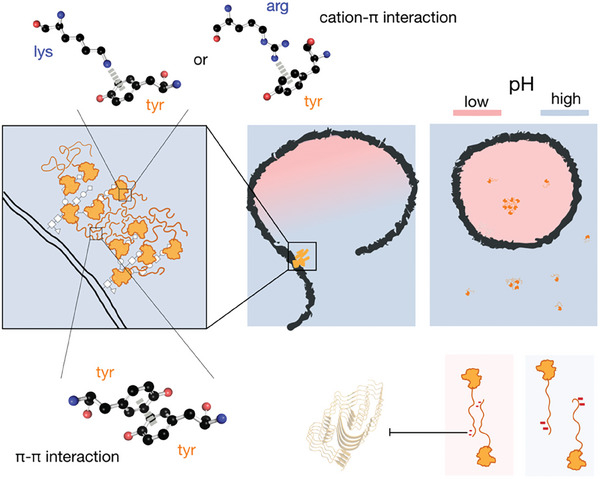
Schematic representation of the proposed galectin‐3 agglutination mechanism. Galectin‐3 agglutinates glycosylated molecules primarily through intermolecular π–π interactions between the disordered N‐terminal domain (NTD) of neighboring molecules^[^ ^31^ ^]^ and cation‐π interactions between the NTD and conserved positively charged residues in the carbohydrate‐recognition domain (CRD). The two negatively charged residues in the NTD regulate self‐assembly at different pHs and prevent aggregation.

Using the IDR of hnRNP‐A1 as a model, Bremer et al. conducted detailed and extensive analyses to understand how various aromatic and charged residues influence IDRs’ phase separation behaviors.^[^ ^51^ ^]^ Consistent with their findings regarding protein polymers with a reduced net charge, our charge‐reduced construct (D3/9G) in the NTD also exhibits a lower saturation concentration, indicating an enhanced propensity for phase transition. However, our study focused not only on the evolutionarily conserved patterns of charged residues but also elucidated the functional importance of the interplay between galectin‐3′s intrinsically disordered NTD and structured CRD. Experiments on twelve carefully chosen mutants revealed that the positively charged residues on the *F*‐face of the CRD interact with the aromatic residues in the disordered NTD through cation‐π interactions, extending our understanding beyond the previously identified π‐π interactions between the NTDs. Intriguingly, the only two charged residues in the NTD (negatively charged) do not significantly contribute to NTD/CRD interactions but rather serve as pH‐responsive elements to prevent rapid fiber growth (**Figure** **7**).

Galectin‐3 is widely recognized as a marker of disrupted endomembrane compartments, such as lysosomes and phagosomes.^[^ ^62^, ^63^, ^64^ ^]^ Several galectins have been identified as “danger signal” sensors for damaged endocytic membranes infected with bacteria.^[^ ^35^, ^65^ ^]^ For example, galectin‐3 and galectin‐8 regulate antibacterial autophagy by detecting specific glycans on compromised intracellular membranes and by recognizing different glycans on leaky intracellular membranes.^[^ ^33^, ^34^ ^]^ A unique feature of galectin‐3 in the galectin family is its IDR,^[^ ^66^ ^]^ which provides an additional regulation mechanism in the form of two aspartates, whose varying protonation states in different pH environments tune galectin‐3′s propensity to self‐assemble. This mechanism would allow galectin‐3 to agglutinate efficiently in the relatively neutral pH environments of lysozyme rupture sites and then detach from their ligands once the lysosome is repaired and the intravesical pH decreases. The negative charges also mitigate the increase in intermolecular NTD/NTD interactions at lower pHs and thereby prevent aggregation (Figure 7), which may also benefit the recycling and endosomal transport of galectin‐3.^[^ ^57^, ^67^ ^]^

The functional significance of these negatively charged residues in the NTD also informs the debate surrounding the differing effects on galectin‐3 nuclear transport of deleting the first 11 residues versus the entire NTD.^[^ ^68^, ^69^ ^]^ Since the first 11 residues include the two aspartates and our findings suggest that this stretch is pivotal in modulating galectin‐3′s propensity to self‐associate and aggregate, removing them is expected to increase self‐assembly, preventing secretion and leading to a loss of nuclear localization.^[^ ^68^ ^]^ On the other hand, although all agglutination‐related functions are lost in the absence of the NTD, CRD‐related nuclear transport should remain unaffected.^[^ ^69^ ^]^ Furthermore, phosphorylation at Ser‐6 or Ser‐12, which adds negative charges, should further attenuate self‐association, and our results also provide a functional rationale for this post‐translational modification.^[^ ^70^, ^71^, ^72^ ^]^

Galectin‐3 is not the only protein involved in endomembrane repair. For example, the ESCRT (endosomal sorting complexes required for transport) complex is recruited to lysosomal repair sites, where it interacts with galectin‐3 through the ALIX protein,^[^ ^36^ ^]^ whose C‐terminal domain interacts with galectin‐3′s NTD.^[^ ^73^ ^]^ Interestingly, ALIX's C‐terminal domain is also an IDR with sparsely charged residues in one part and multiple aromatic residues in the other and has high prion propensity (Figure S8B, Supporting Information). Since ALIX has recently been reported to undergo phase separation,^[^ ^74^ ^]^ it is tempting to propose that local pH variations could also regulate these two IDRs’ heterotypic phase separation propensity and functions, notably in membrane repair.

Our study underscores the critical role of a few charged residues in tuning the assembly processes of prion‐like proteins. By examining galectin‐3, we have highlighted the evolutionary adaptation of charged residues across a broad spectrum of proteins, including FUS, ALIX, and Sup35. This adaptation appears to balance assembly‐related functions while preventing amyloid formation and aggregation, as ≈800 proteins in the human proteome contain prion‐like sequences (listed in the supporting material), most of which are not associated with amyloid fibril‐related diseases. Furthermore, our work may also serve as a precursor to similar investigations for phase separation regulated by the interplay between disordered and structured domains within a protein. Understanding their interactions would be crucial for elucidating the behavior of the prevalent IDR‐tethered proteins in the proteome.

## 4 Experimental Section

### DNA Constructs

Complementary DNA sequences of galectin‐3 variants were integrated into the pHD vector, which includes a hexahistidine‐tagged small ubiquitin‐like modifier protein (6xHis‐SUMO).^[^ ^30^ ^]^ Point mutations were introduced through site‐directed mutagenesis by PCR. The primers used are listed in Table S1 (Supporting Information). All constructs were verified by sequencing (Tri‐I Biotech Inc).

### Protein Expression and Purification

All variants were purified following the established protocol.^[^ ^30^, ^31^ ^]^ Fusion proteins, comprising 6xHis‐SUMO and galectin‐3 variants, were purified using nickel‐charged immobilized metal‐ion affinity chromatography (IMAC) columns (Qiagen). Imidazole was removed through a PD‐10 column (GE Healthcare). Subsequently, 6xHis‐Ulp1^403–621^ protease was introduced into the protein solution at a concentration of 5 µg ml^−1^ for an hour at 4 °C to cleave the 6xHis‐SUMO tag from the galectin‐3 variants. After enzyme digestion, the solution was applied to a nickel‐charged IMAC column again to separate the cleaved components. The flow‐through containing the protein of interest was collected. The collected protein was loaded into a HiLoad Superdex 75 pg gel‐filtration column (GE Healthcare) with an FPLC system or a PD‐10 column to exchange the indicated buffer. Samples were flash‐frozen in liquid nitrogen and stored at −80 °C.

Mutations at the NTD (the full‐length D3G, D9G, and D3/9G) and NTD‐only constructs (NTD, NTD‐D3G, NTD‐D9G, NTD‐D3/9G) required additional purification steps. Full‐length proteins were buffer‐exchanged to pure water (at low concentration) using HiTrap Desalting columns (Cytiva) and subsequently lyophilized. NTD‐only constructs were purified using a C4 reverse‐phase column (Thermo Scientific Inc.), with a gradient elution of acetonitrile (0 to 100% in triple‐distilled water) by an HPLC system before lyophilization. Lyophilized samples were stored in a dry cabinet until use. A protease inhibitor (Roche Applied Science) or 1 mm EDTA (ethylenediaminetetraacetic acid) was added before experiments. The used buffers include 20 mm sodium phosphate or sodium citrate for pH 6.8 and pH 7.0, respectively (no difference in terms of NMR, phase separation, and agglutination studies), 20 mm sodium phosphate at pH 6, 20 mm sodium acetate for pH 5 and 4, and 20 mm sodium citrate at pH 4.

### Bioinformatic Analysis

The levels of intrinsic disorder, prion‐like characteristics, and phase separation propensity were predicted using IUPRED3 or PONDR,^[^ ^40^, ^75^ ^]^ PLAAC,^[^ ^76^ ^]^ and Fuzdrop,^[^ ^55^ ^]^ respectively. The vertebrate proteome was retrieved from the TrEMBL database.^[^ ^77^ ^]^ Homologous sequences were searched using the BLAST algorithm.^[^ ^78^ ^]^ An in‐house Python script is used to execute IUPRED3 or PLAAC on a local computer for proteome‐wide analysis. Phylogenetic relationships were determined using the ETE Toolkit.^[^ ^79^ ^]^ The CRD structures of distantly related vertebrates were modeled using AlphaFold2,^[^ ^80^ ^]^ accessed through the ColabFold interface.^[^ ^81^ ^]^

### Lipopolysaccharide (LPS) Agglutination Assay

LPS from *E. coli* strain O55:B5 was from Merck (Catalog No. L2880). A total amount (*t*) protein of 20 µm was mixed with a final concentration of 0.2 mg mL^−1^ LPS, which was then centrifuged at 10 000x g for 5 min to obtain the supernatant (*s*) and pellet (*p*). The protein amount in the pellet is difficult to determine precisely. Thus, the Bradford assay was used to measure the protein concentration in *t* and *s* samples. In the Bradford assay, bovine serum albumin (BSA) standards and the assay samples were mixed with 170 µL of Bradford reagent, and the absorbance at 594 and 466 nm (A594 and A466) was measured using a TECAN Spark microplate reader. A standard regression line was established from the BSA concentrations and their corresponding A594/A466 ratios for linear calibration.^[^ ^82^ ^]^ Sample concentrations were deduced from this calibrated regression. The protein amount in the pellet is then derived by subtracting *s* from *t*. The “agglutination level” is defined as 1 − [*s*]/[*t*]. The higher value indicates a stronger agglutination ability. All measurements were executed in triplicate.

### Protein Condensation Level Determination

Protein samples were prepared by dissolving the lyophilized protein powder in the specified buffers. The protein solution was then centrifuged at 10 000x g for 60 min to obtain the supernatant (*s*) and the pellet (*p)*. This assay determined the protein sample's tendency to condense at specified buffer conditions and does not distinguish the physical forms of condensates (reversible phase separation, salting out, or irreversible aggregation). Directly determining the protein concentration in the pellet is challenging (difficult to resuspend completely), so the protein amount in the pellet was derived by subtracting the protein concentration *s* from *t*. The concentrations of *t* and *s* were determined by Bradford assay (see above) or by measuring the absorbance at 280 nm. In this type of assay, the “condensation level” is defined as 1 − [*s*]/[*t*]. The higher level indicates more protein samples are in the condensed state.

### Microscopy Analysis

Phase‐separated condensates, agglutinated LPS micelles, and aggregates were imaged under indicated conditions using a Leica DM2500 microscope equipped with a Flexacam C5 camera. A THM120 thermostatic stage (Linkam Scientific Inc.) was used for temperature‐controlled experiments. Aggregation morphology was analyzed at 200 µm protein concentration, with wild‐type aggregates prepared at pH 4.0 and D3/9G constructs at pH 7.0.

### NMR Spectroscopy

NMR data were collected on a Bruker AVIII 850 spectrometer equipped with a TCI cryogenic probe. The ^1^H‐^15^N HSQC spectra and transverse relaxation rate experiments were collected using standard pulse sequences.^[^ ^83^, ^84^, ^85^ ^]^ The transverse relaxation rates were measured with delays of 17, 34, 51, 68, 85, and 102 ms. Peak intensities were fitted to exponential decay with a Monte Carlo procedure to estimate fitting error. The dynamics data were collected in an interleaved manner with an interscan delay of 3 s. All NMR data were collected at 30 °C, unless otherwise stated.

All data were processed using NMRPipe^[^ ^86^ ^]^ and analyzed with SPARKY.^[^ ^87^ ^]^ Peak intensities and errors were determined using the non‐linear line‐shape analysis (nlinLS) function in NMRPipe based on the noise of the spectra. The intensity ratios were normalized to the number of scans. The average chemical shift difference (Δδ^av^) was calculated using:

(1) $$\Delta \delta^{\mathrm{av}}=\sqrt{\frac{{\left(\Delta \delta_{H}\right)}^{2}+{\left(\frac{1}{5}\Delta \delta_{N}\right)}^{2}}{2}}$$

where Δ*δ*_H_ and Δ*δ*_N_ are the differences in chemical shift between two ^1^H‐^15^N HSQC spectra for the amide proton and nitrogen, respectively.

For titration experiments, the dissociation constant (*K*_D_) was derived by fitting the non‐linear regression analysis using the formula:^[^ ^88^ ^]^

(2) $$\Delta \delta^{\mathrm{obs}}=\Delta \delta^{\max}\frac{\left(K_{D}+\left[L\right]+\left[P\right]\right)-\sqrt{{\left(K_{D}+\left[L\right]+\left[P\right]\right)}^{2}-\left(4\left[P\right]\left[L\right]\right)}}{2[P]}$$

where Δ*δ* ^obs^ is the difference in the average chemical shift between free and bound states. Δ*δ* ^max^ is the difference in average chemical shift between free and saturated states. [*P*] and [*L*] are the total concentrations of protein and added ligands.

## Conflict of Interest

The authors declare no conflict of interest.

## Author Contributions

Y.C.S. and T.L.H. contributed equally to this work. J.R.H., T.L.H., and Y.C.S. conceived the project. T.L.H., Y.C.S., C.I.L., W.Y.S., and Y.H.L. performed experiments. J.R.H. performed bioinformatics studies. J.R.H., T.L.H., and Y.C.S. analyzed the results. J.R.H., T.L.H., and Y.C.S wrote the manuscript. All authors approved the final version of the manuscript.

## Supporting information



Supporting Information

## Data Availability

The data that support the findings of this study are available from the corresponding author upon reasonable request.

## References

[1] G. Warren , W. Wickner , Cell 1996, 84, 395. 8608593 10.1016/s0092-8674(00)81284-2

[2] Y. Shin , C. P. Brangwynne , Science 2017, 357, https://www.science.org/toc/science/357/6357. 10.1126/science.aaf4382 28935776

[3] S. F. Banani , H. O. Lee , A. A. Hyman , M. K. Rosen , Nat. Rev. Mol. Cell Biol. 2017, 18, 285s. 10.1038/nrm.2017.7 PMC7434221 28225081

[4] A. A. Hyman , C. A. Weber , F. Julicher , Annu. Rev. Cell Dev. Biol. 2014, 30, 39s. 10.1146/annurev-cellbio-100913-013325 25288112

[5] T. S. Harmon , A. S. Holehouse , M. K. Rosen , R. V. Pappu , Elife 2017, 6, e30294. 29091028 10.7554/eLife.30294 PMC5703641

[6] A. S. Holehouse , R. V. Pappu , Biochemistry 2018, 57, 2415s. 10.1021/acs.biochem.7b01136 PMC6538385 29323488

[7] J. M. Choi , A. S. Holehouse , R. V. Pappu , Annu. Rev. Biophys. 2020, 49, 107s. 10.1146/annurev-biophys-121219-081629 PMC10715172 32004090

[8] A. S. Holehouse , B. B. Kragelund , Nat. Rev. Mol. Cell Biol. 2023. 10.1038/s41580-023-00673-0 PMC11459374 37957331

[9] V. N. Uversky , A. K. Dunker , Biochim. Biophys. Acta 2010, 1804, 1231s. 10.1016/j.bbapap.2010.01.017 PMC2882790 20117254

[10] A. E. Posey , A. S. Holehouse , R. V. Pappu , Methods in enzymology 2018, 611, 1s. 10.1016/bs.mie.2018.09.035 30471685

[11] H. R. Li , T. C. Chen , C. L. Hsiao , L. Shi , C. Y. Chou , J. R. Huang , Biochim. Biophys. Acta 2018, 1866, 214s. 10.1016/j.bbapap.2017.10.001 28988034

[12] C. W. Pak , M. Kosno , A. S. Holehouse , S. B. Padrick , A. Mittal , R. Ali , A. A. Yunus , D. R. Liu , R. V. Pappu , M. K. Rosen , Mol. Cell 2016, 63, 72. 27392146 10.1016/j.molcel.2016.05.042 PMC4973464

[13] D. M. Mitrea , J. A. Cika , C. B. Stanley , A. Nourse , P. L. Onuchic , P. R. Banerjee , A. H. Phillips , C. G. Park , A. A. Deniz , R. W. Kriwacki , Nat. Commun. 2018, 9, 842s. 10.1038/s41467-018-03255-3 PMC5827731 29483575

[14] P. Yang , C. Mathieu , R. M. Kolaitis , P. Zhang , J. Messing , U. Yurtsever , Z. Yang , J. Wu , Y. Li , Q. Pan , J. Yu , E. W. Martin , T. Mittag , H. J. Kim , J. P. Taylor , Cell 2020, 181, 325s. 10.1016/j.cell.2020.03.046 PMC7448383 32302571

[15] S. Sridharan , A. Hernandez‐Armendariz , N. Kurzawa , C. M. Potel , D. Memon , P. Beltrao , M. Bantscheff , W. Huber , S. Cuylen‐Haering , M. M. Savitski , Nat. Chem. Biol. 2022, 18, 1104s. 10.1038/s41589-022-01062-y PMC9512703 35864335

[16] B. Tsang , J. Arsenault , R. M. Vernon , H. Lin , N. Sonenberg , L. Y. Wang , A. Bah , J. D. Forman‐Kay , Proc. Natl. Acad. Sci. 2019, 116, 4218s. 10.1073/pnas.1814385116 PMC6410804 30765518

[17] Y. E. Guo , J. C. Manteiga , J. E. Henninger , B. R. Sabari , A. Dall'Agnese , N. M. Hannett , J. H. Spille , L. K. Afeyan , A. V. Zamudio , K. Shrinivas , B. J. Abraham , A. Boija , T. M. Decker , J. K. Rimel , C. B. Fant , T. I. Lee , I. I. Cisse , P. A. Sharp , D. J. Taatjes , R. A. Young , Nature 2019, 572, 543s. 10.1038/s41586-019-1464-0 PMC6706314 31391587

[18] H. Lu , D. Yu , A. S. Hansen , S. Ganguly , R. Liu , A. Heckert , X. Darzacq , Q. Zhou , Nature 2018, 558, 318s. 10.1038/s41586-018-0174-3 PMC6475116 29849146

[19] S. Wegmann , B. Eftekharzadeh , K. Tepper , K. M. Zoltowska , R. E. Bennett , S. Dujardin , P. R. Laskowski , D. MacKenzie , T. Kamath , C. Commins , C. Vanderburg , A. D. Roe , Z. Fan , A. M. Molliex , A. Hernandez‐Vega , D. Muller , A. A. Hyman , E. Mandelkow , J. P. Taylor , B. T. Hyman , EMBO J. 2018, 37, 98049. 10.15252/embj.201798049 PMC5881631 29472250

[20] M. Saito , D. Hess , J. Eglinger , A. W. Fritsch , M. Kreysing , B. T. Weinert , C. Choudhary , P. Matthias , Nat. Chem. Biol. 2019, 15, 51s. 10.1038/s41589-018-0180-7 30531905

[21] T. J. Nott , E. Petsalaki , P. Farber , D. Jervis , E. Fussner , A. Plochowietz , T. D. Craggs , D. P. Bazett‐Jones , T. Pawson , J. D. Forman‐Kay , A. J. Baldwin , Mol. Cell 2015, 57, 936s. 10.1016/j.molcel.2015.01.013 PMC4352761 25747659

[22] S. Qamar , G. Wang , S. J. Randle , F. S. Ruggeri , J. A. Varela , J. Q. Lin , E. C. Phillips , A. Miyashita , D. Williams , F. Strohl , W. Meadows , R. Ferry , V. J. Dardov , G. G. Tartaglia , L. A. Farrer , G. S. Kaminski Schierle , C. F. Kaminski , C. E. Holt , P. E. Fraser , G. Schmitt‐Ulms , D. Klenerman , T. Knowles , M. Vendruscolo , P. St George‐Hyslop , Cell 2018, 173, 720s. 10.1016/j.cell.2018.03.056 PMC5927716 29677515

[23] M. J. Fossat , A. E. Posey , R. V. Pappu , ChemPhysChem 2023, 24, 202200746s. 10.1002/cphc.202200746 PMC10734359 36599672

[24] M. J. Fossat , A. E. Posey , R. V. Pappu , Biophys. J. 2021, 120, 5438s. 10.1016/j.bpj.2021.11.2886 PMC8715249 34826385

[25] M. J. Fossat , X. Zeng , R. V. Pappu , J. Phys. Chem. B 2021, 125, 4148s. 10.1021/acs.jpcb.1c01073 PMC8154595 33877835

[26] P. Dogra , A. Joshi , A. Majumdar , S. Mukhopadhyay , J. Am. Chem. Soc. 2019, 141, 20380s. 10.1021/jacs.9b10892 31783713

[27] S. Alberti , A. Gladfelter , T. Mittag , Cell 2019, 176, 419s. 10.1016/j.cell.2018.12.035 PMC6445271 30682370

[28] T. M. Franzmann , M. Jahnel , A. Pozniakovsky , J. Mahamid , A. S. Holehouse , E. Nuske , D. Richter , W. Baumeister , S. W. Grill , R. V. Pappu , A. A. Hyman , S. Alberti , Science 2018, 10.1126/science.aao5654. 29301985

[29] M. R. King , K. M. Ruff , A. Z. Lin , A. Pant , M. Farag , J. M. Lalmansingh , T. Wu , M. J. Fossat , W. Ouyang , M. D. Lew , E. Lundberg , M. D. Vahey , R. V. Pappu , Cell 2024, 187, 1889s. 10.1016/j.cell.2024.02.029 PMC11938373 38503281

[30] Y. H. Lin , D. C. Qiu , W. H. Chang , Y. Q. Yeh , U. S. Jeng , F. T. Liu , J. R. Huang , J. Biol. Chem. 2017, 292, 17845s. 10.1074/jbc.M117.802793 PMC5663883 28893908

[31] Y. P. Chiu , Y. C. Sun , D. C. Qiu , Y. H. Lin , Y. Q. Chen , J. C. Kuo , J. R. Huang , Nat. Commun. 2020, 11, 1229s. 10.1038/s41467-020-15007-3 PMC7060198 32144274

[32] Z. Zhao , X. Xu , H. Cheng , M. C. Miller , Z. He , H. Gu , Z. Zhang , A. Raz , K. H. Mayo , G. Tai , Y. Zhou , Proc. Natl. Acad. Sci. 2021, 118, e2021074118. 33952698 10.1073/pnas.2021074118 PMC8126832

[33] I. C. Weng , H. L. Chen , T. H. Lo , W. H. Lin , H. Y. Chen , D. K. Hsu , F. T. Liu , Glycobiology 2018, 28, 392s. 10.1093/glycob/cwy017 29800364

[34] M. H. Hong , W. H. Lin , I. C. Weng , Y. H. Hung , H. L. Chen , H. Y. Chen , P. Chen , C. H. Lin , W. Y. Yang , F. T. Liu , Glycobiology 2019, 30, 49s. 10.1093/glycob/cwz075 31553041

[35] S. Banfer , R. Jacob , Biomolecules 2020, 10, 1232. 32847140 10.3390/biom10091232 PMC7563435

[36] J. Jia , A. Claude‐Taupin , Y. Gu , S. W. Choi , R. Peters , B. Bissa , M. H. Mudd , L. Allers , S. Pallikkuth , K. A. Lidke , M. Salemi , B. Phinney , M. Mari , F. Reggiori , V. Deretic , Dev. Cell 2020, 52, 69s. 10.1016/j.devcel.2019.10.025 PMC6997950 31813797

[37] M. L. Fermino , C. D. Polli , K. A. Toledo , F. T. Liu , D. K. Hsu , M. C. Roque‐Barreira , G. Pereira‐da‐Silva , E. S. Bernardes , L. Halbwachs‐Mecarelli , PLoS One 2011, 6, e26004s. 10.1371/journal.pone.0026004 PMC3198732 22031821

[38] Y. Li , M. Komai‐Koma , D. S. Gilchrist , D. K. Hsu , F. T. Liu , T. Springall , D. Xu , J. Immunol. 2008, 181, 2781s. 10.4049/jimmunol.181.4.2781 18684969

[39] T. Zarin , B. Strome , G. Peng , I. Pritisanac , J. D. Forman‐Kay , A. M. Moses , Elife 2021, 10, e60220. 33616531 10.7554/eLife.60220 PMC7932695

[40] G. Erdos , M. Pajkos , Z. Dosztanyi , Nucleic Acids Res. 2021, 49, W297s. 10.1093/nar/gkab408 PMC8262696 34048569

[41] J. M. Otaki , M. Tsutsumi , T. Gotoh , H. Yamamoto , J. Chem. Inf. Model. 2010, 50, 690s. 10.1021/ci900452z 20210310

[42] A. K. Dunker , J. D. Lawson , C. J. Brown , R. M. Williams , P. Romero , J. S. Oh , C. J. Oldfield , A. M. Campen , C. M. Ratliff , K. W. Hipps , J. Ausio , M. S. Nissen , R. Reeves , C. Kang , C. R. Kissinger , R. W. Bailey , M. D. Griswold , W. Chiu , E. C. Garner , Z. Obradovic , J Mol Graph Model 2001, 19, 26s. 10.1016/s1093-3263(00)00138-8 11381529

[43] J. Yan , J. Cheng , L. Kurgan , V. N. Uversky , Cell. Mol. Life Sci. 2020, 77, 2423s. 10.1007/s00018-019-03292-1 PMC11105052 31486849

[44] W. L. Ho , J. R. Huang , Protein Sci. 2022, 31, e4317s. 10.1002/pro.4317 PMC9045073 35481633

[45] S. S. Patel , B. J. Belmont , J. M. Sante , M. F. Rexach , Cell 2007, 129, 83s. 10.1016/j.cell.2007.01.044 17418788

[46] R. Zangi , R. Zhou , B. J. Berne , J. Am. Chem. Soc. 2009, 131, 1535s. 10.1021/ja807887g 19123816

[47] T. Akerud , E. Thulin , R. L. Van Etten , M. Akke , J. Mol. Biol. 2002, 322, 137s. 10.1016/s0022-2836(02)00714-3 12215420

[48] O. K. Baryshnikova , B. D. Sykes , Protein Sci. 2006, 15, 2568s. 10.1110/ps.062255806 PMC2242403 17075134

[49] M. R. Jensen , S. M. Kristensen , C. Keeler , H. E. Christensen , M. E. Hodsdon , J. J. Led , Proteins 2008, 73, 161s. 10.1002/prot.22039 18409193

[50] J. P. Brady , P. J. Farber , A. Sekhar , Y. H. Lin , R. Huang , A. Bah , T. J. Nott , H. S. Chan , A. J. Baldwin , J. D. Forman‐Kay , L. E. Kay , Proc. Natl. Acad. Sci. 2017, 114, E8194s. 10.1073/pnas.1706197114 PMC5625912 28894006

[51] A. Bremer , M. Farag , W. M. Borcherds , I. Peran , E. W. Martin , R. V. Pappu , T. Mittag , Nat. Chem. 2022, 14, 196s. 10.1038/s41557-021-00840-w PMC8818026 34931046

[52] S. C. Ng , D. Gorlich , Nat. Commun. 2022, 13, 6172s. 10.1038/s41467-022-33697-9 PMC9579204 36257947

[53] M. Dzuricky , B. A. Rogers , A. Shahid , P. S. Cremer , A. Chilkoti , Nat. Chem. 2020, 12, 814s. 10.1038/s41557-020-0511-7 PMC8281385 32747754

[54] S. Roberts , M. Dzuricky , A. Chilkoti , FEBS Lett. 2015, 589, 2477s. 10.1016/j.febslet.2015.08.029 PMC4599720 26325592

[55] M. Hardenberg , A. Horvath , V. Ambrus , M. Fuxreiter , M. Vendruscolo , Proc. Natl. Acad. Sci. 2020, 117, 33254s. 10.1073/pnas.2007670117 PMC7777240 33318217

[56] T. von Mach , M. C. Carlsson , T. Straube , U. Nilsson , H. Leffler , R. Jacob , Biochem. J. 2014, 457, 107s. 10.1042/BJ20130933 24147723

[57] T. Straube , T. von Mach , E. Honig , C. Greb , D. Schneider , R. Jacob , Traffic 2013, 14, 1014s. 10.1111/tra.12086 23710780

[58] A. Patel , H. O. Lee , L. Jawerth , S. Maharana , M. Jahnel , M. Y. Hein , S. Stoynov , J. Mahamid , S. Saha , T. M. Franzmann , A. Pozniakovski , I. Poser , N. Maghelli , L. A. Royer , M. Weigert , E. W. Myers , S. Grill , D. Drechsel , A. A. Hyman , S. Alberti , Cell 2015, 162, 1066s. 10.1016/j.cell.2015.07.047 26317470

[59] K. A. Burke , A. M. Janke , C. L. Rhine , N. L. Fawzi , Mol. Cell 2015, 60, 231s. 10.1016/j.molcel.2015.09.006 PMC4609301 26455390

[60] F. Luo , X. Gui , H. Zhou , J. Gu , Y. Li , X. Liu , M. Zhao , D. Li , X. Li , C. Liu , Nat. Struct. Mol. Biol. 2018, 25, 341s. 10.1038/s41594-018-0050-8 29610493

[61] J. Wang , J. M. Choi , A. S. Holehouse , H. O. Lee , X. Zhang , M. Jahnel , S. Maharana , R. Lemaitre , A. Pozniakovsky , D. Drechsel , I. Poser , R. V. Pappu , S. Alberti , A. A. Hyman , Cell 2018, 174, 688s. 10.1016/j.cell.2018.06.006 PMC6063760 29961577

[62] S. Aits , J. Kricker , B. Liu , A. M. Ellegaard , S. Hamalisto , S. Tvingsholm , E. Corcelle‐Termeau , S. Hogh , T. Farkas , A. Holm Jonassen , I. Gromova , M. Mortensen , M. Jaattela , Autophagy 2015, 11, 1408s. 10.1080/15548627.2015.1063871 PMC4590643 26114578

[63] W. P. Flavin , L. Bousset , Z. C. Green , Y. Chu , S. Skarpathiotis , M. J. Chaney , J. H. Kordower , R. Melki , E. M. Campbell , Acta Neuropathol. 2017, 134, 629s. 10.1007/s00401-017-1722-x 28527044

[64] J. J. Siew , H. M. Chen , H. Y. Chen , H. L. Chen , C. M. Chen , B. W. Soong , Y. R. Wu , C. P. Chang , Y. C. Chan , C. H. Lin , F. T. Liu , Y. Chern , Nat. Commun. 2019, 10, 3473s. 10.1038/s41467-019-11441-0 PMC6677843 31375685

[65] T. L. Thurston , M. P. Wandel , N. von Muhlinen , A. Foeglein , F. Randow , Nature 2012, 482, 414s. 10.1038/nature10744 PMC3343631 22246324

[66] G. A. Rabinovich , M. A. Toscano , Nat. Rev. Immunol. 2009, 9, 338s. 10.1038/nri2536 19365409

[67] D. Schneider , C. Greb , A. Koch , T. Straube , A. Elli , D. Delacour , R. Jacob , Eur. J. Cell Biol. 2010, 89, 788s. 10.1016/j.ejcb.2010.07.001 20705359

[68] H. C. Gong , Y. Honjo , P. Nangia‐Makker , V. Hogan , N. Mazurak , R. S. Bresalier , A. Raz , Cancer Res. 1999, 59, 6239s. 10626818

[69] J. C. Gaudin , B. Mehul , R. C. Hughes , European Cell Biology Organization 2000, 92, 49s. 10.1016/S0248-4900(00)88763-8 10761697

[70] F. T. Liu , R. J. Patterson , J. L. Wang , Biochim. Biophys. Acta 2002, 1572, 263s. 10.1016/s0304-4165(02)00313-6 12223274

[71] E. A. Cowles , N. Agrwal , R. L. Anderson , J. L. Wang , J. Biol. Chem. 1990, 265, 17706s. 2170392

[72] M. E. Huflejt , C. W. Turck , R. Lindstedt , S. H. Barondes , H. Leffler , J. Biol. Chem. 1993, 268, 26712s. 8253806

[73] S. F. Wang , C. H. Tsao , Y. T. Lin , D. K. Hsu , M. L. Chiang , C. H. Lo , F. C. Chien , P. Chen , Y. M. Arthur Chen , H. Y. Chen , F. T. Liu , Glycobiology 2014, 24, 1022s. 10.1093/glycob/cwu064 PMC4181451 24996823

[74] R. D. Elias , Y. Zhu , Q. Su , R. Ghirlando , J. Zhang , L. Deshmukh , Sci. Adv. 2023, 9, eadg3913s. 10.1126/sciadv.adg3913 PMC10348681 37450591

[75] Z. Obradovic , K. Peng , S. Vucetic , P. Radivojac , A. K. Dunker , Proteins 2005, 61, 176s. 10.1002/prot.20735 16187360

[76] A. K. Lancaster , A. Nutter‐Upham , S. Lindquist , O. D. King , Bioinformatics 2014, 30, 2501s. 10.1093/bioinformatics/btu310 PMC4147883 24825614

[77] C. UniProt , Nucleic Acids Res. 2023, 51, D523s. 10.1093/nar/gkac1052 PMC9825514 36408920

[78] R. Zaru , S. Orchard , C. UniProt , Curr. Protoc. 2023, 3, e697s. 10.1002/cpz1.697 PMC10034637 36943033

[79] J. Huerta‐Cepas , F. Serra , P. Bork , Mol. Biol. Evol. 2016, 33, 1635s. 10.1093/molbev/msw046 PMC4868116 26921390

[80] J. Jumper , R. Evans , A. Pritzel , T. Green , M. Figurnov , O. Ronneberger , K. Tunyasuvunakool , R. Bates , A. Zidek , A. Potapenko , A. Bridgland , C. Meyer , S. A. A. Kohl , A. J. Ballard , A. Cowie , B. Romera‐Paredes , S. Nikolov , R. Jain , J. Adler , T. Back , S. Petersen , D. Reiman , E. Clancy , M. Zielinski , M. Steinegger , M. Pacholska , T. Berghammer , S. Bodenstein , D. Silver , O. Vinyals , et al., Nature 2021, 596, 583s. 10.1038/s41586-021-03819-2 PMC8371605 34265844

[81] M. Mirdita , K. Schutze , Y. Moriwaki , L. Heo , S. Ovchinnikov , M. Steinegger , Nat. Methods 2022, 19, 679s. 10.1038/s41592-022-01488-1 PMC9184281 35637307

[82] T. Zor , Z. Selinger , Anal. Biochem. 1996, 236, 302s. 10.1006/abio.1996.0171 8660509

[83] M. Piotto , V. Saudek , V. Sklenar , J. Biomol. NMR 1992, 2, 661s. 10.1007/BF02192855 1490109

[84] G. Bodenhausen , D. J. Ruben , Chem. Phys. Letters 1980, 69, 185s.

[85] N. A. Farrow , R. Muhandiram , A. U. Singer , S. M. Pascal , C. M. Kay , G. Gish , S. E. Shoelson , T. Pawson , J. D. Forman‐Kay , L. E. Kay , Biochemistry 1994, 33, 5984s. 10.1021/bi00185a040 7514039

[86] F. Delaglio , S. Grzesiek , G. W. Vuister , G. Zhu , J. Pfeifer , A. Bax , J. Biomol. NMR 1995, 6, 277s. 10.1007/BF00197809 8520220

[87] T. D. Goddard , D. G. Kneller , Sparky, Vol. 3, University of California, San Francisco, 2005.

[88] N. J. Baxter , T. H. Lilley , E. Haslam , M. P. Williamson , Biochemistry 1997, 36, 5566s. 10.1021/bi9700328 9154941

