# Supplementary material for: A Few Charged Residues in Galectin‐3′s Folded and Disordered Regions Regulate Phase Separation
Source: Adv Sci (Weinh). 2024 Sep 9;11(41):2402570. doi: 10.1002/advs.202402570 (PMC11538691; doi:10.1002/advs.202402570)
Supplement: Supplementary file 1 — Supporting Information [file ADVS-11-2402570-s001.pdf]

ADVANCED SCIENCE
Open Access

Supporting Information

for *Adv. Sci.*, DOI 10.1002/advs.202402570

A Few Charged Residues in Galectin-3′s Folded and Disordered Regions Regulate Phase Separation

*Yung-Chen Sun, Tsung-Lun Hsieh, Chia-I Lin, Wan-Yu Shao, Yu-Hao Lin and Jie-rong Huang**

Supporting Information

A few charged residues in galectin-3's folded and disordered regions regulate phase separation

*Yung-Chen Sun, Tsung-Lun Hsieh, Chia-I Lin, Wan-Yu Shao, Yu-Hao Lin, and Jie-rong Huang**

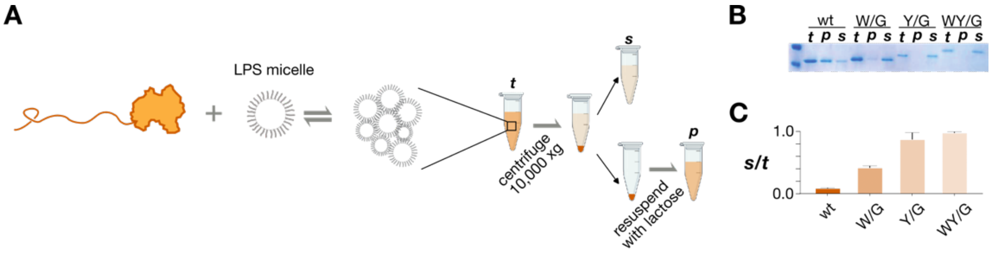

Figure S1. An example of using SDS-PAGE to quantify the level of agglutination. (A) The flowchart of the experiments. We used the previously published constructs as examples. W/G, Y/G, WY/G: replacing 2 tryptophans or 10 tyrosines or both in the N-terminal domain NTD with glycines. (B) A typical SDS-PAGE of the total amount of loaded sample (*t*), those in the pellet (*p*), and in the supernatant (*s*) after 10,000 xg centrifugation for 5 min. (C) The level of agglutination can be quantified by verifying the intensities on the SDS-PAGE gel or using the Bradford assay to quantify the amount of protein in the supernatant. The amount in the supernatant or the pellet is normalized by the total proteins, i.e., *s/t* or *p/t,* throughout this article.

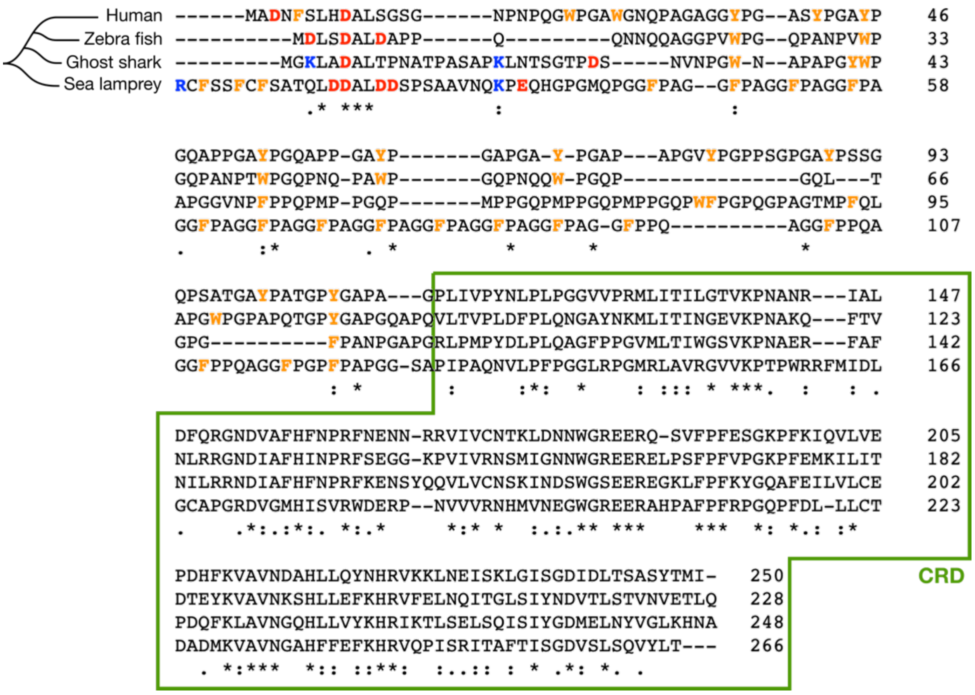

Figure S2. Sequence alignment of galectins with an intrinsically disordered region (IDR). The sequences of the most distant vertebrates to humans (*Homo sapiens*, P17931) are compared: zebrafish (*Danio rer*io, Q6TGN4), ghost shark (*Callorhinchus milii*, A0A4W3GAP9), sea lamprey (*Petromyzon marinus*, S4RBU8). The structured carbohydrate-recognition domain (CRD) is indicated in the green box. Within the IDR, the positively charged lysine (K) and arginine (R), negatively charged aspartate (D) and glutamate (E), and aromatic tryptophan (W), tyrosine (Y), and phenylalanine (F) are colored with blue, red, and orange.

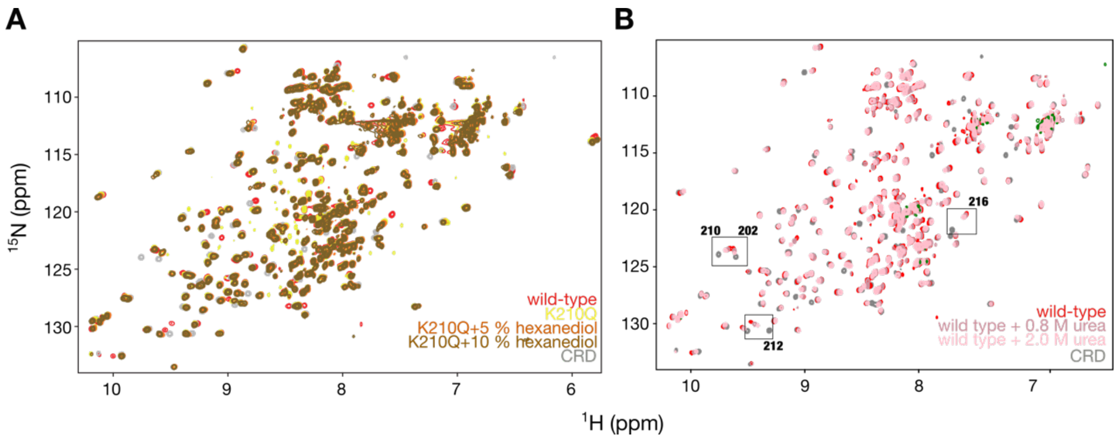

Figure S3. (A) The overlaid ^{1}H-^{15}N HSQC spectra of the wild-type (red), K210Q (yellow), and K210Q with additional 5% (orange), 10% (brown) 1,6-hexanediol, and the CRD-only contracts (grey). (B) The overlaid ^{1}H-^{15}N HSQC spectra of the wild-type (red), with additional 0.8 (pink), 2.0 M urea (light pink), and CRD-only constructs (gray). Notable residues, including residue 216 and others on the *F*-face, are indicated.

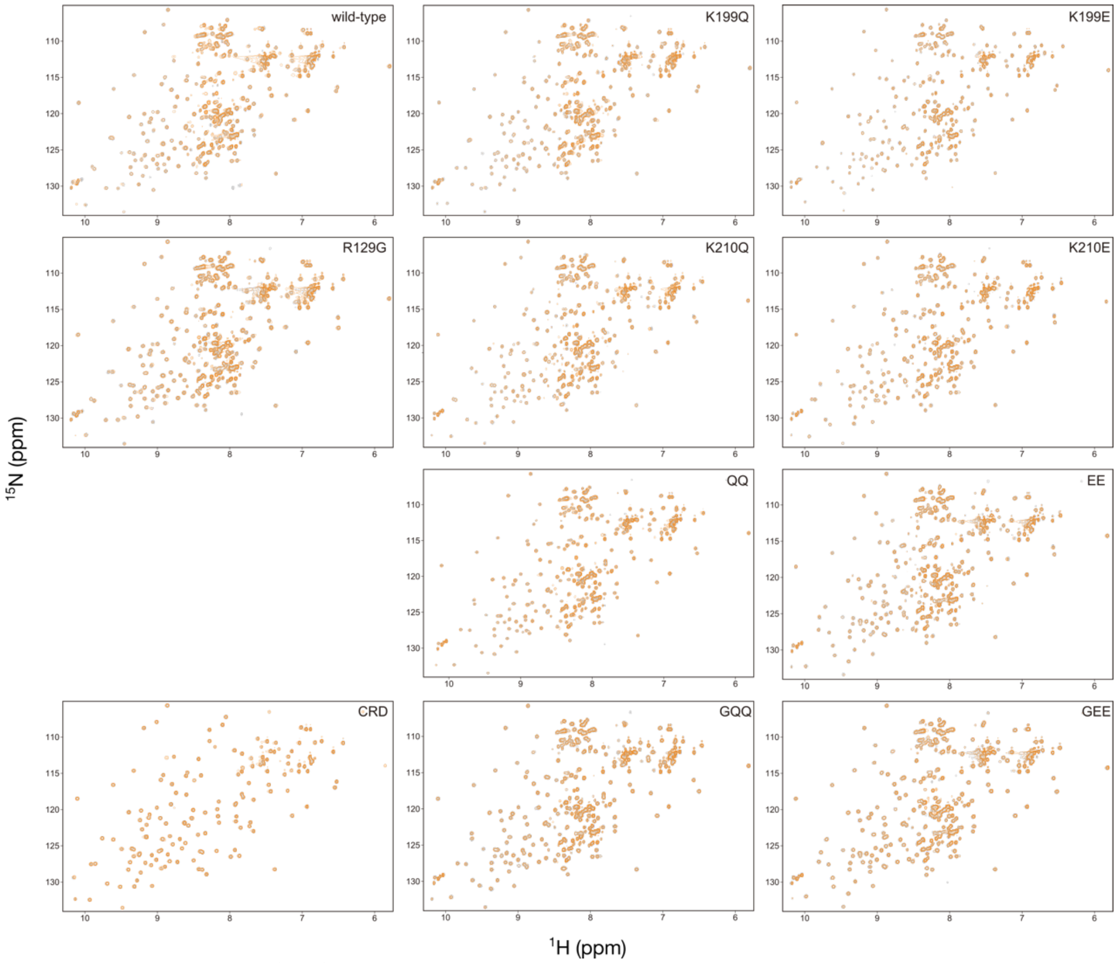

Figure S4. The overlaid ^{1}H-^{15}N HSQC spectra of all the mutations at 400 μM (orange) and 40 μM (gray).

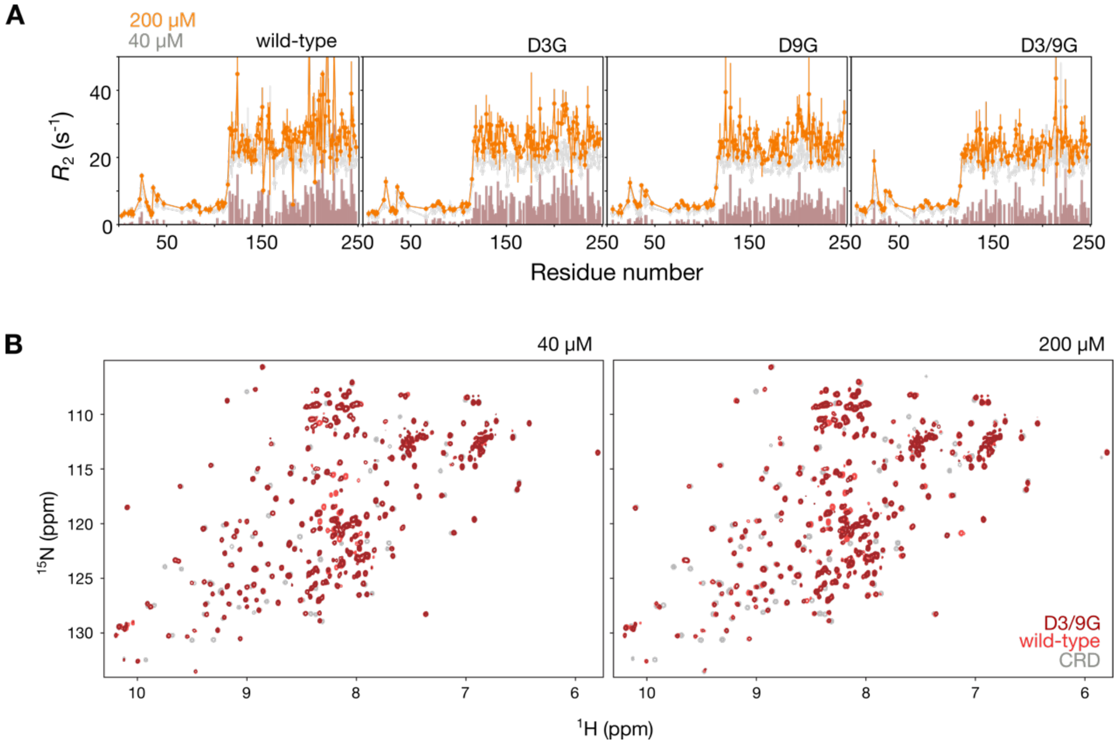

Figure S5. (A) The transverse relaxation rate constants (R_2) of the wild-type, D3G, D9G, and D3/9G in 200 µM (orange) and 40 µM (gray), and their difference (brown bars). (B) The overlaid ^{1}H-^{15}N HSQC spectra of D3/9G, the wild-type, and CRD-only in 40 µM or 200 µM.

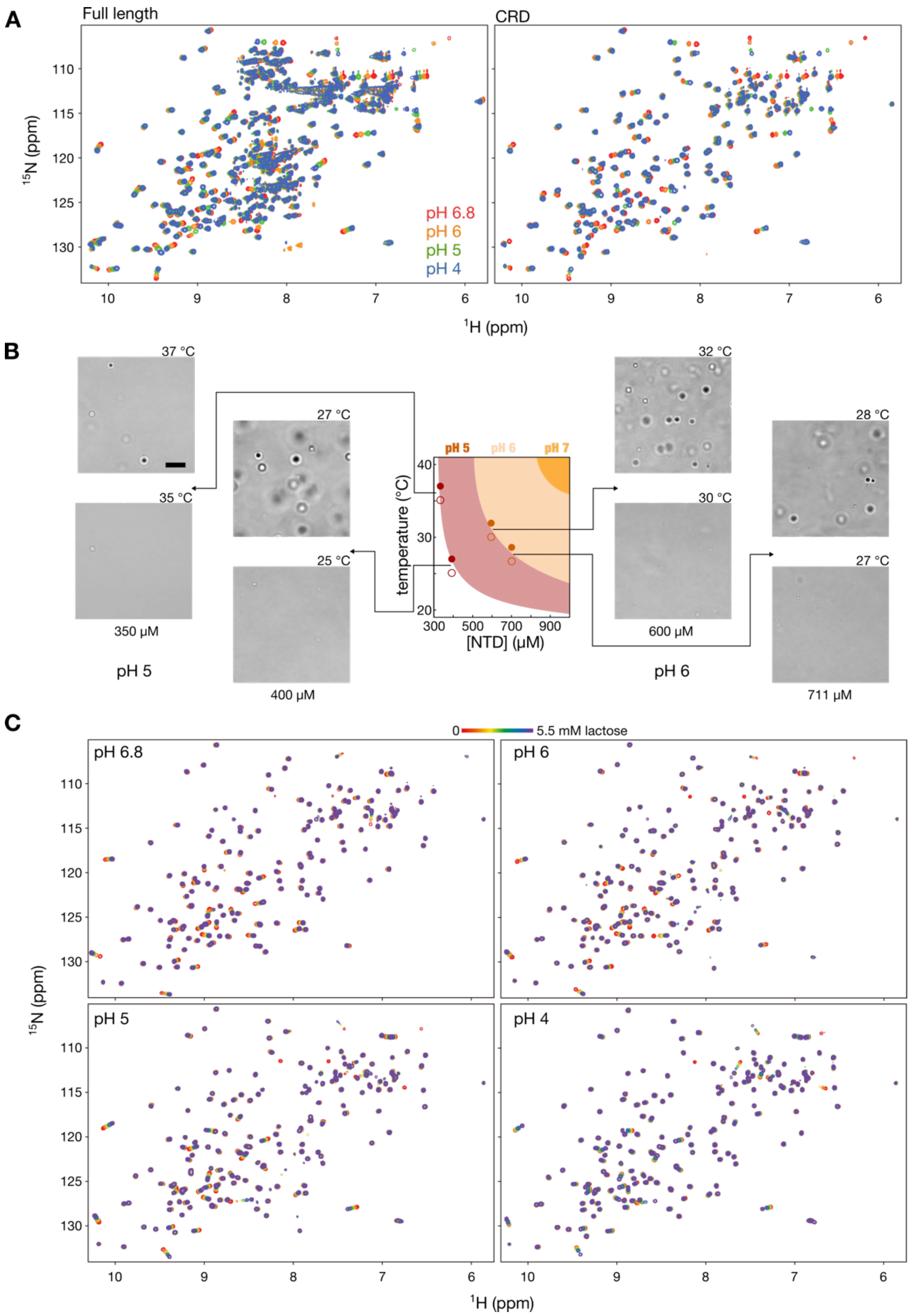

Figure S6. (A) The ^{1}H-^{15}N HSQC spectra of pH titration for the full-length and carbohydrate-recognition domain (CRD) of galectin-3. (B) The phase separation temperatures for galectin-3's NTD at pH 5 and pH 6 across varying concentrations. Scale bar: 10 μm. (C) Lactose titration of galectin-3's CRD under four different pH conditions.

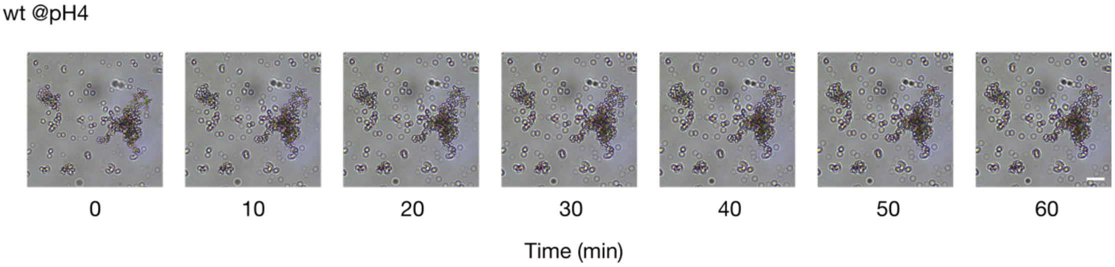

Figure S7. Time-lapse bright-field microscopic images of the aggregation process for the wild-type at pH 4 (scale bar: 10 µm). Compared to the D3/G mutant at pH 7 (Fig. 6G), the aggregates did not extend over the same period.

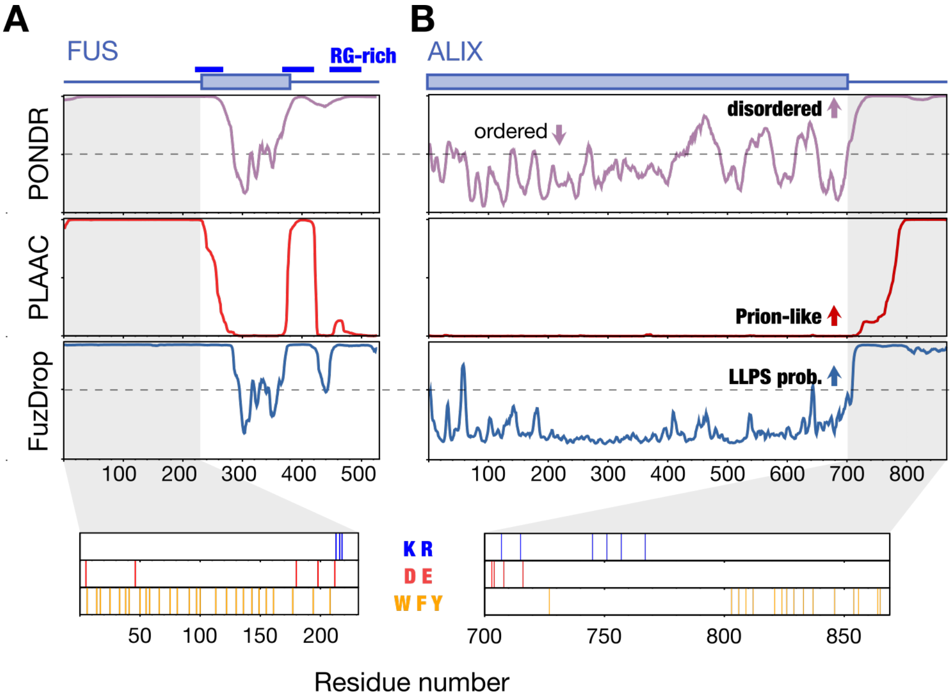

Figure S8. The sequence analysis for proteins with intrinsically disordered regions having similar properties as galectin-3. (A) The protein FUS; (B) The protein ALIX. Annotations and color schemes follow those in Figure 2B.

Table S1. Primers used in this work.

Construct	Template	Primer Sequence (5'-3')
K199E	pHD-Gal3-wt	*Fw:* GGGAAACCATTCGAAATACAAGTACTGGTTGAACC<br>*Rv:* CAGTACTTGTATTTCGAATGGTTTCCCACTTTCAA
K199Q	pHD-Gal3-wt	*Fw:* AGTGGGAAACCATTCCAGATACAAGTA<br>*Rv:* TTCAACCAGTACTTGTATCTGGAATGG
R129G	pHD-Gal3-wt	*Fw:* GGAGTGGTGCCTGGCATGCTGATAACAATTCTGGG<br>*Rv:* TGTTATCAGCATGCCAGGCACCACTCCCCCAGGCA
K210E	pHD-Gal3-wt	*Fw:* GGAGTGGTGCCTGGCATGCTGATAACAATTCTGGG<br>*Rv:* TGTTATCAGCATGCCAGGCACCACTCCCCCAGGCA
K210Q	pHD-Gal3-wt	*Fw:* CCTGACCACTTCCAGGTTGCAGTGAATGATGCTCA<br>*Rv:* ATTCACTGCAACCTGGAAGTGGTCAGGTTCAACCA
EE	pHD-Gal3-K210E	*Fw:* GGGAAACCATTCGAAATACAAGTACTGGTTGAACC<br>*Rv:* CAGTACTTGTATTTCGAATGGTTTCCCACTTTCAA
QQ	pHD-Gal3-K210Q	*Fw:* AGTGGGAAACCATTCCAGATACAAGTA<br>*Rv:* TTCAACCAGTACTTGTATCTGGAATGG
GEE	pHD-Gal3-EE	*Fw:* GGAGTGGTGCCTGGCATGCTGATAACAATTCTGGG<br>*Rv:* TGTTATCAGCATGCCAGGCACCACTCCCCCAGGCA
GQQ	pHD-Gal3-QQ	*Fw:* GGAGTGGTGCCTGGCATGCTGATAACAATTCTGGG<br>*Rv:* TGTTATCAGCATGCCAGGCACCACTCCCCCAGGCA
D3G	pHD-Gal3-wt	*Fw:* GGCATGGCAGGCAATTTTTCG<br>*Rv:* AGATAACGCGCCATGGAGCGA
D9G	pHD-Gal3-wt	*Fw:* GGCATGGCAGGCAATTTTTCG<br>*Rv:* AGATAACGCGCCATGGAGCGA
D3/9G	pHD-Gal3-wt	*Fw:* GGCATGGCAGGCAATTTTTCG<br>*Rv:* AGATAACGCGCCATGGAGCGA

wt: the wild-type; EE: K199E/K210E; QQ: K199Q/K210Q;

GEE: R129G/K199E/K210E; GQQ: R129G/K199Q/K210Q
